# Baicalin Promotes Mammary Gland Development via Steroid-Like Activities

**DOI:** 10.3389/fcell.2021.682469

**Published:** 2021-07-06

**Authors:** Weizhen Chen, Wei Wei, Liya Yu, Xin Zhang, Fujing Huang, Qiping Zheng, Lingli Wang, Cheguo Cai

**Affiliations:** ^1^Department of Orthopaedics, Frontier Science Center for Immunology and Metabolism, Zhongnan Hospital of Wuhan University, Medical Research Institute, Wuhan University, Wuhan, China; ^2^Guangzhou University of Chinese Medicine, Mathematical Engineering Academy of Chinese Medicine, Guangzhou, China; ^3^Jiangsu Key Laboratory of Medical Science and Laboratory Medicine, Department of Hematological Laboratory Science, School of Medicine, Jiangsu University, Zhenjiang, China; ^4^Shenzhen Academy of Peptide Targeting Technology at Pingshan, Shenzhen Tyercan Bio-pharm Co., Ltd., Shenzhen, China; ^5^Dongguan and Guangzhou University of Chinese Medicine Cooperative Academy of Mathematical Engineering for Chinese Medicine, Dongguan City, China; ^6^Shenzhen Beike Biotechnology Co., Ltd., Shenzhen, China

**Keywords:** baicalin, mammary stem cell, mammary development, breast cancer, steroid hormone

## Abstract

Baicalin, the main flavonoid component extracted from *Scutellaria* roots, has a variety of biological activities and is therefore used in the treatment of many kinds of diseases. However, whether baicalin affects the normal development of tissues and organs is still unclear. Here, using a mouse mammary gland model, we investigated the effects of baicalin on the expansion of mammary stem cells (MaSCs) and mammary development, as well as breast cancer progression. Interestingly, we found that baicalin administration significantly accelerates duct elongation at puberty, and promotes alveolar development and facilitates milk secretion during pregnancy. Furthermore, self-renewal of MaSCs was significantly promoted in the presence of baicalin. Moreover, in a tumor xenograft model, baicalin promoted tumor growth of the MDA-MB-231 cell line, but suppressed tumor growth of the ZR-751 cell line. Mechanistically, baicalin can induce expression of the protein C receptor, while inhibiting the expression of the estrogen receptor. Transcriptome analysis revealed that baicalin is involved in signaling pathways related to mammary gland development, immune response, and cell cycle control. Taken together, our results from comprehensive investigation of the biological activity of baicalin provide a theoretical basis for its rational clinical application.

## Introduction

**FIGURE 1:**
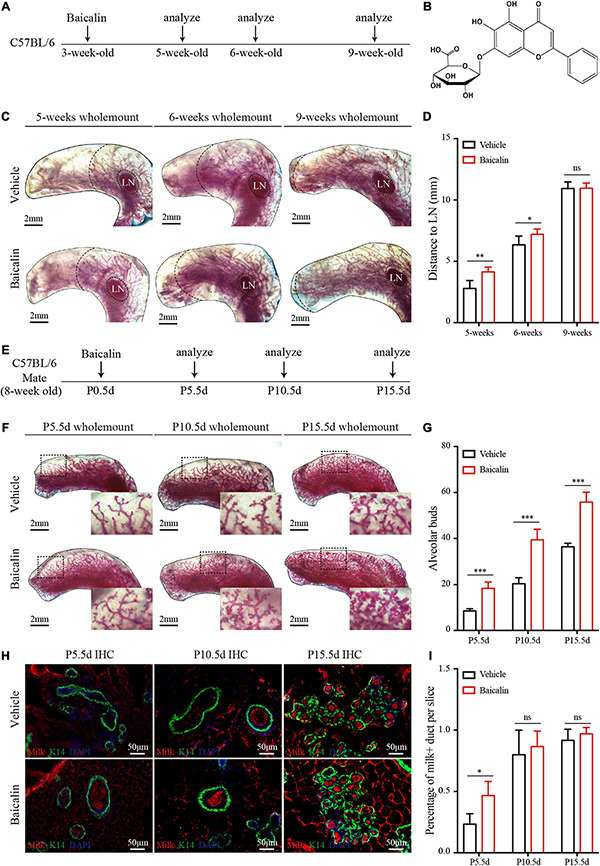
Baicalin accelerates mammary development during puberty and pregnancy. **(A)** Illustration of the baicalin stimulation strategy using C57BL/6 mice from puberty to adulthood. The mice were treated with baicalin at the start of puberty, when the mice were 3 weeks old. The mammary glands were harvested from 5-, 6-, and 9-week-old mice. **(B)** The chemical structure of baicalin. **(C)** Wholemount carmine staining (red) of mammary glands isolated from 3- to 9-week-old female mice. The data represent three independent experiments. **(D)** Quantification of duct elongation indicates that baicalin accelerates the elongation of mammary gland ducts at 5 and 6 weeks, but not at 9 weeks. Student’s *t*-test: **P < 0.01; *P < 0.05; ns, not significant. **(E)** Illustration of the baicalin stimulation strategy using C57BL/6 mice during pregnancy. To study the role of baicalin in pregnancy, pregnant 8-week-old mice were treated with baicalin at day 0.5 of pregnancy (P0.5d) every day until sample collection. The mammary glands were harvested at P5.5d, P10.5d, and P15.5d. **(F)** Wholemount carmine staining (red) of mammary glands isolated from pregnant female mice. The black box indicates the focal structure. The data represent three independent experiments. **(G)** Quantification of alveolar cells indicates that baicalin increases the formation of alveolar cells at P5.5d, P10.5d, and P15.5d. Student’s *t*-test: ***P < 0.001; ns, not significant. **(H)** Images of sections from pregnant mice showing milk secretion. Scale bar, 50 μm. **(I)** Quantification of milk secretion indicates that baicalin can induce the production of milk at P5.5d, but not at P10.5d and P15.5d. Student’s *t*-test: * *P* < 0.05; ns, not significant.

Baicalin (7-glucuronic acid, 5,6-dihydroxy-flavone, Figure 1B) is a flavonoid monomer extracted from the root of *Scutellaria baicalensis*, a Traditional Chinese Medicine (TCM) (Zhang et al., 2017). Previous studies have demonstrated that baicalin can attenuate the progress of certain of diseases and relieve pain. Baicalin has also been reported to participate in many kinds of biological processes, and is therefore used as a therapeutic for a variety of diseases including cancer. For example, it can initiate reprogramming of tumor-associated macrophages into M1-like macrophages and promote the production of pro-inflammatory cytokines, thereby suppressing the growth of hepatocellular carcinoma tumors (Tan et al., 2015); it can up-regulate *DEPP* expression and induce cellular senescence in human colon cancer (Wang Z. et al., 2018). Baicalin is also used in the treatment of other diseases. It can promote osteoblast differentiation through Wnt/β-catenin signal transduction, and is used in the treatment of post-menopausal osteoporosis (Guo et al., 2011). It can attenuate hyperglycemia-induced malformation of the cardiovascular system (Wang G. et al., 2018), activate hepatic CPT1 to ameliorate diet-induced obesity and hepatic steatosis (Dai et al., 2018), and ameliorate lupus autoimmunity (Yang et al., 2019). It also can inhibit the TGF-β1-mediated epithelial-mesenchymal transition, cell growth, and migration of human breast cancer cells (Chung et al., 2015). However, these previous studies on baicalin mainly focused on pathological states. The regulatory effect of baicalin on the physiological state of organisms remains unclear. Moreover, a systematic understanding of the molecular signals induced by baicalin is lacking.

Because of its accessibility and unique postnatal development, the mammary gland is an excellent model for studying the development of tissues and organs and adult stem cell behavior (Stingl et al., 2006; Van Keymeulen et al., 2011; Zhao et al., 2017; Chakrabarti et al., 2018). It is an epithelial organ and is composed of a branching network of ducts and lobuloalveolar structures. There are two primary epithelial cell lineages: myoepithelial (basal, Lin^–^CD29^*hi*^CD24^+^) and luminal (Lin^–^CD29^*lo*^CD24^+^) cells (Shackleton et al., 2006). Mammary gland development is accomplished postnatally and is governed by the concerted action of systemic hormones and local growth factors (Fu et al., 2019). Estrogen and progesterone are two important mitogens of mammary cells at puberty and during pregnancy (Feng et al., 2007; Brisken and Ataca, 2015), and their receptors are expressed in mammary luminal cells. Estrogen signaling regulates mammary ductal elongation at puberty (Mallepell et al., 2006), and progesterone signaling induces side-branching of the mammary gland duct and contributes to alveologenesis during pregnancy (Brisken et al., 1998). MaSCs are located in the mammary basal layer and have the ability to self-renew as well as to differentiate into luminal cells, which is critical for mammary gland maintenance and regeneration. Wnt/β-catenin signaling is one of the most important local growth factors that promote the self-renewal of mammary stem cells (MaSCs) (Badders et al., 2009; Zeng and Nusse, 2010; van Amerongen et al., 2012). MaSCs can be labeled by the protein C receptor (Procr), which is encoded by the target gene of the Wnt/β-catenin signaling pathway (Wang et al., 2015). *In vitro* expansion of MaSCs has always been difficult because they tend to differentiate in the absence of proper self-renewing signals. Zeng et al. established a three-dimensional (3D) culture system, and found that MaSCs cultured in this system can maintain self-renewal for a long time in the presence of Wnt3a (Zeng and Nusse, 2010). Subsequently, Cai et al. (2014) found that Wnt4 and Rspo1 can maintain the proliferation of MaSCs *in vivo*, and that Wnt4 and Rspo1 are regulated by estrogen (E2) and progesterone (PG). Based on this finding, they established a culture system using E2 and PG to expand MaSCs *in vitro* (Cai et al., 2014). However, Wnt3a purification has proven difficult. In addition, combined E2 and PG treatment easily causes side effects, which makes it not conducive to clinical application. Therefore, it is important to look for new alternative and economic methods to expand MaSCs *in vitro*.

Although baicalin, which is a flavonoid, has been reported to have estrogen-like biological activity and can activate the Wnt signaling pathway (Guo et al., 2011; Zhang et al., 2015), its roles in stem cell biology and in regulating organ development have rarely been reported. Moreover, like most TCMs, a comprehensive understanding of the molecular mechanism underlying baicalin function is still lacking. In this study, using a mammary gland model, we investigated the estrogen-like biological function of baicalin and its underlying mechanism in the regulation of MaSC self-renewal, mammary development, and breast cancer oncogenesis. The results from our study not only provide insights into the mechanism by which baicalin regulates MaSC self-renewal and mammary development, but also provide exciting opportunities for the development of baicalin as a treatment for mammary dysplasia caused by hormone secretion disorders.

## Materials and Methods

### Experimental Animals

C57BL/6, Actin-DsRed (Jackson Laboratories), and nude strains were used in this study. All of the mice (3 weeks old) were purchased from Nanjing Biomedical Research Institute of Nanjing University and were maintained in a specific-pathogen-free (SPF) animal facility. Experimental procedures were approved by the Animal Care and Use Committee of Wuhan University.

### Reagents and Determination of the Baicalin Dose

Baicalin (∼95% purity) was purchased from Sigma-Aldrich, Co. (St. Louis, United States), and dissolved in sterile 0.9% NaCl when it was used. According to previous results (Zhang et al., 2015; Huang et al., 2016; Wang Z. et al., 2018; Yang et al., 2019), 50–200 mg/kg body weight (BW) of baicalin administered via intraperitoneal injection has a significant effect on the physiological and pathological states of mouse models. In our pretest, we intraperitoneally injected different doses (50, 100, and 200 mg/kg BW) of baicalin into 3-week-old C57 mice and found that 100 mg/kg BW of baicalin could increase mammary gland development without side effects. Thus, we chose 100 mg/kg BW of baicalin for the mammary development model. In addition, in our pretest we also found that 200 mg/kg BW of baicalin could promote tumor growth of the MDA-MB-231 cell line, but significantly suppressed tumor growth of the ZR-75-1 cell line without side effects. Therefore, 200 mg/kg BW of baicalin was useful for the breast cancer model. In the 3D culture model, 100 μM baicalin could significantly increase the number and size of basal cell colonies, and reduced the colony size and number of luminal cells. Therefore, 100 μM baicalin was chosen for the 3D culture model.

### Baicalin Administration

Thirty-six C57BL/6 mice (3 weeks old) were randomly divided into two groups. One group was intraperitoneally injected with 100 mg/kg BW baicalin every day for 6 weeks, and the other group was injected with an equal amount of vehicle as a control. The fourth mammary glands of 5-, 6-, and 9-week-old mice were harvested. In addition, 36 pregnant females (C57BL/6) were weighed and randomly allocated into control and experimental groups at P0.5d. The experimental group was intraperitoneally injected with 100 mg/kg baicalin every day, and the control group was injected with an equal amount of vehicle until P15.5d. The fourth mammary gland samples were harvested at P5.5d, P10.5d, and P15.5d.

### Ovariectomy and Baicalin Replacement

Ovariectomies were conducted on Actin-DsRed mice (3 weeks old) using a conventional protocol. In brief, the mice were unilaterally ovariectomized and allowed to recover for 1 week. Baicalin (100 mg/kg BW) was then intraperitoneally injected every day. After 3 and 5 weeks, mammary glands were harvested for analysis.

### Primary Cell Preparation

Mammary glands were isolated from 6- to 9-week-old virgin or other female mice at specified stages. Minced tissue was placed in culture medium [RPMI 1640 with 25 mM HEPES, 5% fetal bovine serum (FBS), 1% penicillin-streptomycin-glutamine, 300 U/ml collagenase III (Worthington)] and digested for 2 h at 37°C. After lysis of the red blood cells in NH_4_Cl, a single-cell suspension was obtained by sequential incubation with 0.25% trypsin-EDTA at 37°C for 5 min and 0.1 mg/ml DNase I (Sigma) for 5 min with gentle pipetting, followed by filtration through 70-μm cell strainers. The total primary mammary gland epithelial cells that were obtained were cultured in DMEM high glucose medium (Hyclone) supplemented with 10% FBS, 100 units/ml streptomycin, 100 units/ml penicillin, and 0.3 mg/ml L-glutamine at 37°C and 5% CO_2_. The medium was changed every day until cell collection.

### Cell Labeling and Flow Cytometry

For cell labeling, the following antibodies were used: FITC-conjugated CD31, CD45, and TER119 (Pharmingen^TM^), CD24-PE/cy7 (Biolegend), CD29-APC (Biolegend), Procr (eBioscience), and Sca1 (eBioscience). Antibodies were incubated on ice for 20 min in PBS with 5% FBS. Before cell sorting and analysis, cells were filtered through 40-μm strainers. All cell sorting assays were performed using a FACS Aria (BD, United States). All analyses were performed using an LSRFortessaX20 (BD, United States). The purity of sorted cell populations was routinely checked and ensured to be more than 95%.

### *In vitro* Colony Formation Assay

FACS-sorted cells were resuspended at a density of 4 × 10^5^ cells per milliliter in chilled 100% growth factor-reduced Matrigel (BD Bioscience), and the mixture was allowed to polymerize before covering with culture medium [DMEM/F12; ITS (1:100; Sigma); 50 ng/ml EGF; and either DMSO or 100 μM baicalin]. Culture medium was changed every 24 h. Primary colony numbers were scored after 6–7 days in culture. For passaging colonies, the medium was aspirated, and Matrigel was digested by incubation in 500 μl of Matrigel recovery solution (BD Bioscience) for 1 h on ice. Colonies released from Matrigel were harvested after centrifuging. Cells were obtained through incubation with 0.25% Trypsin-EDTA for 5 min at 37°C followed by gentle pipetting. Cells were then replated in Matrigel as described above. In assays examining the requirement for baicalin in maintaining stem cell properties, baicalin was withdrawn for 5 days in secondary culture prior to transplantation.

### Mammary Fat Pad Transplantation and Analysis

The basal colonies were resuspended in 50% Matrigel in PBS with 50% FBS, and 0.04% Trypan Blue (Sigma) and 10 μl of the mixture was injected into the cleared fat pads of 3-week-old nude females. Reconstituted mammary glands were harvested 6–10 weeks after surgery. Outgrowths were detected under a dissection microscope (Leica) after carmine staining. Outgrowths with > 10% of the host fat pad filled were scored as positive.

### Cell Culture

Eph4, MDA-MB-231, ZR-75-1, and 293T cells were cultured in DMEM-high glucose medium (Hyclone) supplemented with 10% FBS, 100 units/ml streptomycin, 100 units/ml penicillin, and 0.3 mg/ml L-glutamine at 37°C and 5% CO_2_. The medium was changed every day, and cells were passaged every 2 days.

### RT-qPCR

Total RNA was extracted using RNAiso plus (Takara), and the PrimeScript RT Master Mix Kit (Takara) with oligo (dT) primers was used for the reverse transcription reaction. RT-qPCR was performed using a CFX (Bio-Rad) with a FastStart Universal SYBR Green Master Mix Kit (Roche). *Gapdh* served as an internal control. The reaction mixtures were incubated at 95°C for 10 min, followed by 39 cycles of 15 s at 95°C and 30 s at 60°C. Primers used in this study are listed in Table 1.

**TABLE 1:** Primers designed for real-time RT-qPCR.

**Name**	**RefSeqID**	**Sense primer (5′–3′)**	**Antisense primer (5′–3′)**	**Amplicon (bp)**
*Gapdh*	NM_008085	TGGATTTGGACGCATTGGTC	TTTGCACTGGTACGTGTTGAT	211
*Procr*	NM_011171	AATGCCTACAACCGGACTCG	ACCAGTGATGTGTAAGAGCGA	131
*Esr1*	NM_007956	CCTCCCGCCTTCTACAGGT	CACACGGCACAGTAGCGAG	128
*Elf5*	NM_010125	ATGTTGGACTCCGTAACCCAT	GCAGGGTAGTAGTCTTCATTGCT	104
*Socs2*	NM_001168657	TGCGGATTGAGTACCAAGATGG	CTGTCCGTTTATCCTTGCACA	132
*Bax*	NM_007527	TGAAGACAGGGGCCTTTTTG	AATTCGCCGGAGACACTCG	140
*Pgr*	NM_008829	CTCCGGGACCGAACAGAGT	ACAACAACCCTTTGGTAGCAG	122
*Areg*	NM_009704	GGTCTTAGGCTCAGGCCATTA	CGCTTATGGTGGAAACCTCTC	137
*GAPDH*	NM_001256799	GGAGCGAGATCCCTCCAAAAT	GGCTGTTGTCATACTTCTCATGG	197
*PROCR*	NM_006404	CCTACAACCGCACTCGGTATG	CGCGGAAATATGTTTCTGCACA	80
*ERS1*	NM_000125	CCCACTCAACAGCGTGTCTC	CGTCGATTATCTGAATTTGGCCT	180

### Western Blot Analysis

Cells were lysed with ice-cold RIPA lysis buffer. Proteins (30 μg) were resolved by SDS-PAGE and transferred to polyvinylidene fluoride membranes. Immunoblots were developed in chemiluminescence reagent (PerkinElmer Life Sciences) and exposed in a dark room. The primary antibodies used were Procr (abcam, ab151403), ER (Merck, #06-935), and beta-actin (Sigma, A2228), and the second antibodies used were horseradish peroxidase-conjugated mouse (CST, 7076S) and rabbit (CST, 7074S).

### Immunofluorescence

Frozen sections were prepared by air-drying and fixing for 5 min in cold 4% paraformaldehyde. Tissue sections were incubated with primary antibodies (K8, 1:500, DSHB; K14, 1:1,000, Dia-An Biotechnology Co., Ltd; Milk, 1:500, Nordic Immunological Laboratories) at 4°C overnight, followed by three washes with PBST. Tissue sections were incubated with secondary antibodies for 1 h at 25°C, and slides were counterstained with DAPI (Thermo Fisher Scientific). Images were taken by a fluorescence microscope (ZEISS, Germany).

### Tumor Models and Analysis

All animal studies were approved by the Wuhan University Animal Care and Use Committee. Breast cancer cell lines MDA-MB-231 (TNBC subtype) and ZR-75-1 (Luminal breast cancer subtype) were transplanted into the fourth fat pad of adult nude mice. Tumors were measured every 3 days with a caliper, and tumor volume was calculated using the following formula: Tumor volume = width × width × length × 0.52. When tumors reached 50–100 mm^3^, mice were divided into groups for treatment. Baicalin was injected at a concentration of 200 mg/kg BW every day, and the control group was injected with an equal amount of vehicle. The treatment was terminated when the tumor volume reached 1,000 mm^3^.

### Luciferase Assay

The *Procr* and *Esr1* promoters were separately cloned into the pGL4.17 vector to generate the luciferase reporters (Geng et al., 2020). 293T cells were plated into 24-well tissue culture dishes. After 16–20 h, 293T cells were co-transfected with 0.33 μg firefly luciferase reporter plasmid (pGL4.17) containing the promoter region of *Procr* or *ER*, 0.033 μg Renilla luciferase plasmid (pRL-TK), and a specific amount of baicalin per well of the 24-well plates. After 24 h, the cells were lysed and luciferase activities were measured using the Dual-Luciferase Reporter Assay System (Promega, Madison, United States). The experiment was repeated three times.

### RNA Sequencing and Data Processing

The 3D-cultured basal and luminal cells were harvested. Total RNA was extracted using RNAiso plus (Takara). mRNA was then enriched by oligo (dT) beads. RNA-seq libraries were generated using the KAPA Stranded RNA-seq Kit for Illumina with multiplexing primers, according to the manufacturer’s protocol. Then sequencing was performed on an Illumina Nova sequencer.

Reference genome and gene model annotation files were downloaded from the Ensembl genome website directly (Folder: Mus_musculus.GRCm38.94.chr.fa). We selected HISAT2 as the mapping tool. The number of perfect clean tags for each gene was calculated and then normalized in fragments per kilobases per million mapped Reads (FPKM) by the software of featureCounts v1.5.0. Genes with an FPKM > 0 in all samples were retained for further analysis. Genes were defined as differentially expressed genes (DEGs) if the *P*-value of the computed *t*-test was < 0.05 and the log_2_ fold change in expression was > 1. Heatmaps were generated using R.

GSEA was performed to examine whether the genes identified as DEGs for each cell type were members of specific signaling pathways. GO analysis of DEGs was implemented using the GOseq R package, in which gene length bias was corrected. GO terms with a *P*-value less than 0.05 were considered significantly enriched. KEGG analysis is used to understand high-level functions and utilities of a biological system from molecular-level information and other high-throughput experimental technologies. We used GSEA 4.1.0 software to test the statistical enrichment of DEGs in KEGG pathways.

### Statistical Analysis

All of the results are presented as mean ± SD unless otherwise stated. Differences were considered significant when *P* < 0.05 in an unpaired Student’s *t*-test in Prism 6.0. Three independent experiments were carried out for statistical analysis unless specified otherwise.

## Results

### Baicalin Promotes Mammary Duct Elongation at Puberty and Alveolar Development During Pregnancy

There are two key stages of postnatal mammary development: one at puberty, mainly driven by E2, and the other during pregnancy, mainly driven by PG. To understand the regulatory effects of baicalin during mammary development, we first studied its effect at puberty; 3-week-old female wild-type mice were intraperitoneally injected with baicalin. The fourth mammary glands were taken from mice at 5, 6, and 9 weeks of age for carmine staining (Figure 1A). The results of wholemount carmine staining indicated that the ducts of the baicalin group at 5 and 6 weeks were significantly longer than those of the control group, but there was no significant difference at 9 weeks of age, as determined by measuring the distance from the end of the duct to the lymph node (LN) (Figures 1C,D). In the presence of baicalin, there was no difference in the formation of lateral branches (data not shown).

To observe the effects of baicalin during pregnancy, baicalin was injected intraperitoneally on day 0.5 of pregnancy (P0.5d), and then once a day until tissue sample collection. Carmine staining was performed on P5.5d, P10.5d, and P15.5d (Figure 1E). The results of carmine staining indicated that there were significantly more alveoli in the baicalin group than in the control group at P5.5d, P10.5d, and P15.5d (Figures 1F,G). To understand the effect of baicalin on mammary function, we measured milk secretion during pregnancy. Immunofluorescence analysis indicated that milk secretion occurred significantly earlier in the baicalin treatment group than in the control group (Figures 1H,I). Together, these data indicated that baicalin promotes the development of the mammary gland at two critical stages. Of note, baicalin can also promote the secretion of milk.

### Baicalin Induces Duct Growth and Lateral Branching in an Ovariectomized Mouse Model

**FIGURE 2:**
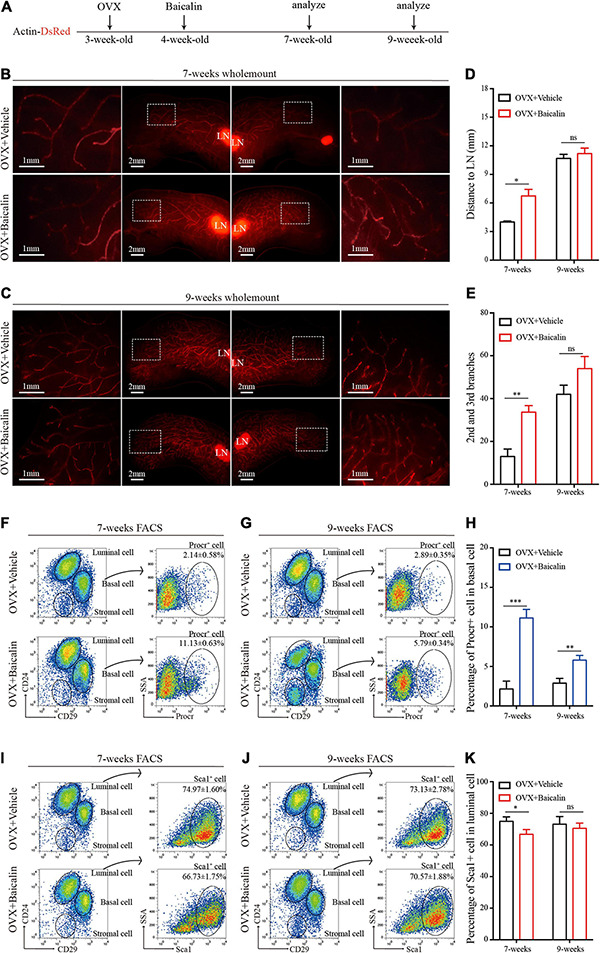
Baicalin accelerates mammary development in an ovariectomized model. **(A)** Illustration of the baicalin treatment strategy using Actin-DsRed ovariectomized (OVX) mice. Four-week-old OVX mice were treated with baicalin, and mammary glands were harvested from 7- and 9-week-old mice. **(B,C)** Wholemount staining (red) of mammary glands isolated at from 7-week-old **(B)** and 9-week-old **(C)** female mice. The white box indicates the focal structure. The data represent three independent experiments. **(D)** Quantification of mammary gland duct length indicates that baicalin accelerates the elongation of the mammary gland duct in 7-week-old mice. Student’s *t*-test: *P < 0.05; ns, not significant. **(E)** Quantification of lateral branches indicates that baicalin increases the formation of the second and third side branches of mammary glands in 7- but not 9-week-old mice (ns). Student’s *t*-test: **P < 0.01; ns, not significant. **(F,G)** FACS analysis of mammary epithelial cells revealed the percentages of Procr^+^ basal cells in 7-week-old **(F)** and 9-week-old **(G)** mice. **(H)** Quantification analysis indicates that baicalin increases the number of Procr^+^ basal cells in 7- and 9-week-old mice. Student’s *t*-test: ***P < 0.001; **P < 0.01. **(I,J)** FACS analysis of mammary epithelial cells revealed the percentages of Sca1^+^ luminal cells in 7-week-old **(I)** and 9-week-old **(J)** mice. **(K)** Quantification analysis indicates that baicalin decreases the number of Sca1^+^ luminal cells in 7-week-old mice. Student’s *t*-test: * *P* < 0.05; ns, not significant.

We have shown that baicalin can promote mammary development in wild-type mice. To test whether baicalin can replace endogenous ovarian hormones to maintain mammary development, we further investigated the effect of baicalin in an ovariectomized Actin-DsRed mouse model (Figure 2A). Baicalin was injected intraperitoneally, and the fourth pair of mammary glands were examined in mice at 7 and 9 weeks of age by monitoring fluorescence. FACS analysis was used to analyze the mammary epithelial cell population. We observed that baicalin significantly increased the formation of the second and third grade duct branches in 7- and 9-week-old mice, and promoted significantly more duct elongation in 7-week-old mice, as illustrated by wholemount imaging and statistical analysis (Figures 2B–E). However, in 9-week-old mice, the mammary glands of the control group were all extended, filling the fat pad, similar to those of the baicalin-treated group (Figures 2C–E). Together, these data further strengthen the hypothesis that baicalin promotes mammary gland development and plays a role similar to that of endogenous steroid hormones.

### Baicalin Promotes Proliferation of Procr-Labeled MaSCs *in vivo*

The hormone signal-responsive cells labeled with Sca1 located in the mammary luminal layer are responsible for receiving hormone signals that regulate mammary gland development. Procr-labeled MaSCs exist in the mammary basal layer and maintain the stability of the mammary epithelial cell populations. Both types of cells play an important role in the development of the mammary gland (Shehata et al., 2012; Wang et al., 2015). To understand how baicalin promotes the elongation of the mammary duct and the formation of lateral branches, we further investigated the regulatory effect of baicalin on Sca1-labeled mammary luminal cells and Procr-labeled MaSCs. FACS analysis indicated that in the presence of baicalin, the number of Procr-labeled MaSCs (Lin^–^CD29^*hi*^CD24^+^Procr^+^) of 7- and 9-week-old mice increased significantly (Figures 2F–H), whereas the number of Sca1-labeled luminal cells (Lin^–^CD29^*lo*^CD24^+^Sca1^+^) decreased slightly (Figures 2I–K). Taken together, the results suggest that baicalin can promote the proliferation of Procr-labeled MaSCs in an ovariectomized mouse model.

### Baicalin Promotes the Expansion of MaSCs and Inhibits Proliferation of Luminal Cells *in vitro*

**FIGURE 3:**
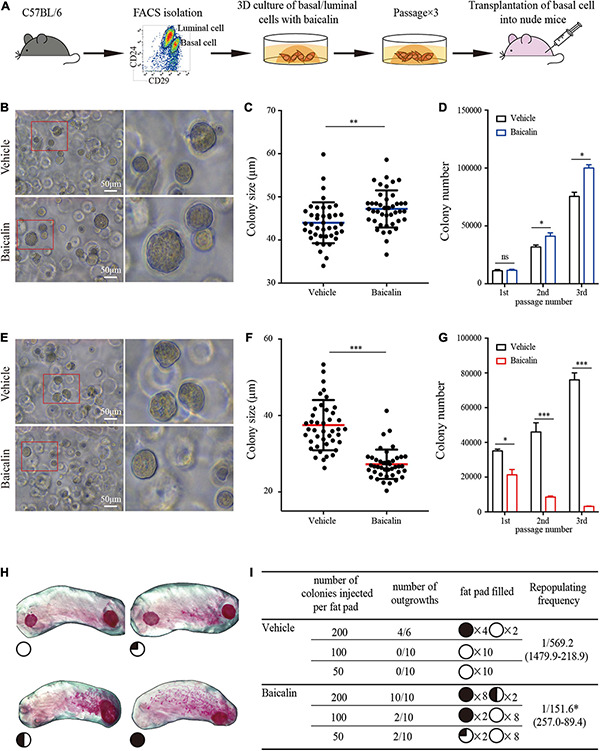
Baicalin promotes the colony formation ability and regeneration capacity of basal cells. **(A)** Illustration of the use of FACS to obtain basal and luminal cells, 3D culturing of basal and luminal cells under baicalin treatment, and basal cell transplantation assays. **(B)** The colony formation efficiency of basal cells after treatment with baicalin in Matrigel culture. Scale bar, 50 μm. The red box indicates the focal structure. The data represent three independent experiments. **(C)** Colony size of basal cells in Matrigel culture. Student’s *t*-test: **P < 0.01. **(D)** Number of basal cell colonies after three passages. Student’s *t*-test: *P < 0.05; ns, not significant. **(E)** The colony formation efficiency of luminal cells after treatment with baicalin in Matrigel culture. Scale bar, 50 μm. The red box indicates the focal structure. The data represent three independent experiments. **(F)** Colony size of luminal cells in Matrigel culture. Student’s *t*-test: ***P < 0.001. **(G)** Colony number of luminal cells after three passages. Student’s *t*-test: ***P < 0.001; *P < 0.05. **(H)** Representative images of fat pads with different percentages of filling. **(I)** The numbers and sizes (shown as the percentage of fat pad filled) of mammary outgrowths are combined from three independent experiments.

Expanding stem cells *in vitro* is important for regeneration medicine but has been a challenge. In view of the fact that baicalin can promote the proliferation of MaSCs *in vivo*, it is of great interest to determine whether baicalin can promote the self-renewal of MaSCs *in vitro*, and whether it can be used as a growth factor to promote the expansion of MaSCs. Next, we used baicalin to expand MaSCs *in vitro*. We also investigated the effect of baicalin on breast luminal cells *in vitro*. Basal cells or luminal cells were cultured in matrix gel in the presence of baicalin. Colony size and numbers of basal and luminal cells were measured after consecutive rounds of passaging to evaluate their colony forming ability. Basal cell colonies in the third passage were collected and transplanted into the cleared fat pads of immunocompromised recipients by gradient dilution (Figure 3A). We observed that baicalin significantly increased the number of basal cell colonies in serial passages, and the colony size of basal cells was also significantly increased (Figures 3B–D). On the contrary, baicalin significantly reduced the colony size and number of luminal cells (Figures 3E–G). This is consistent with the *in vivo* experimental results. To test whether the cells proliferated *in vitro* maintain the characteristics of stem cells during the process of expansion, we transplanted the cell colonies from the third passage into cleared fat pads to assess mammary outgrowth. We observed that in the vehicle group, there was no mammary outgrowth when 50 and 100 colonies were transplanted, but there was mammary outgrowth when 200 colonies were transplanted, with a repopulating frequency of 1/569.2. However, the amplified colonies from the baicalin-treated group showed effective reconstitution ability; when 50 colonies were transplanted, there was a small amount of outgrowth, and when 100 and 200 colonies were transplanted, there was efficient mammary reconstitution, with a repopulating frequency of 1/151.6 (Figures 3H,I). Together, these results indicated that baicalin promotes the proliferation of MaSCs and maintains their properties, but inhibits the proliferation of luminal cells.

### *Procr* Expression Is Up-Regulated in the Presence of Baicalin

**FIGURE 4:**
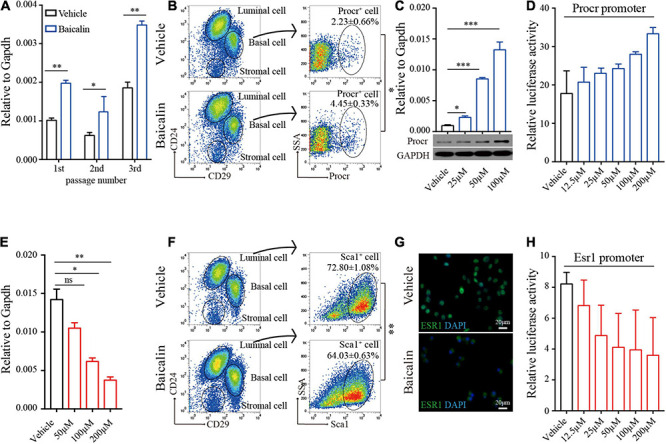
Baicalin up-regulates *Procr* expression and down-regulates *Esr1* expression. **(A)** RT-qPCR analyses were performed to detect *Procr* expression in basal cells after different numbers of serial passages. Data are presented as mean ± SD from three independent experiments. Student’s *t*-test: **P < 0.01; *P < 0.05. **(B)** FACS of mammary epithelial cells to determine the percentage of Procr + basal cells in C57BL/6 mice. Student’s *t*-test: *P < 0.05. **(C)** RT-qPCR and western blot analyses were performed to detect *Procr* expression in primary cells treated with baicalin. Data are presented as mean ± SD from three independent experiments. Student’s *t*-test: ***P < 0.001; *P < 0.05. **(D)** Luciferase assays of *Procr* promoter activity. Data are presented as mean ± SD from three independent experiments. **(E)** RT-qPCR analyses were performed to detect *Esr1* expression in luminal cells. Data are presented as mean ± SD from three independent experiments. Student’s *t*-test: **P < 0.01; *P < 0.05; ns, not significant. **(F)** FACS of mammary epithelial cells to determine the percentage of Sca1^+^ luminal cells in C57BL/6 mice. Student’s *t*-test: **P < 0.01. **(G)** Immunostaining images of ESR1 protein in ZR-75-1 cells upon treatment with baicalin. Scale bar, 20 μm. **(H)** Luciferase assays of *Esr1* promoter activity. Data are presented as mean ± SD from three independent experiments.

As shown above, baicalin can promote the expansion of MaSCs labeled with Procr both *in vivo* (ovariectomized model) and *in vitro*. To determine whether this is due to baicalin-mediated up-regulation of *Procr* expression, we first measured *Procr* expression in cultured basal cells treated with baicalin. Quantitative real-time PCR (RT-qPCR) analysis indicated that *Procr* mRNA levels were significantly up-regulated in the first, second, and third passages of basal cells in the presence of baicalin (Figure 4A). Then, we investigated the expansion of Procr-labeled MaSCs in wild-type mice. FACS analysis indicated that the number of Procr-labeled MaSCs was also significantly increased after baicalin administration (Figure 4B). Next, we investigated whether baicalin up-regulates *Procr* expression in a dose-dependent manner. Primary mammary cells were cultured in the presence of various doses of baicalin for 48 h. Western blot and RT-qPCR analyses indicated that baicalin up-regulated the expression of *Procr* in a dose-dependent manner (Figure 4C). To investigate whether baicalin regulates the transcription of *Procr*, we utilized a luciferase reporter driven by the mouse *Procr* proximal promoter (2.0 kb upstream of the first ATG). The results showed that baicalin induced luciferase expression in a dose-dependent manner (Figure 4D). Together, these data indicated that *Procr* expression is up-regulated by baicalin, and that baicalin directly promotes the transcription of the *Procr* gene.

### *Esr1* Expression Is Down-Regulated Upon Baicalin Treatment

FACS analysis detected a slightly decreased number of Sca1-labeled luminal cells (Esr1^+^/Pgr^+^) in the ovariectomized model after baicalin administration. Similar results were obtained in FACS analysis of the wild-type mouse model (Figure 4F). Since the luminal cells labeled with Sca1 are known to express *Esr1* (Shehata et al., 2012), these results suggest that *Esr1* expression may be down-regulated by baicalin. To determine whether baicalin downregulates *Esr1* and causes a decrease in the number of Sca1-labeled luminal cells, we examined *Esr1* expression in cultured luminal cells treated with baicalin. RT-qPCR analysis indicated that baicalin significantly reduced the expression of *Esr1* in a dose-dependent manner (Figure 4E). Notably, in the control group ESR1 was mainly located in the nucleus, while in the baicalin-treated group ESR1 localized to the cytoplasm (Figure 4G). To investigate whether baicalin regulates the transcription of *Esr*1, we utilized a luciferase reporter driven by the proximal promoter of mouse *Esr1* (Geng et al., 2020). We found that luciferase activity was down-regulated by baicalin in a dose-dependent manner (Figure 4H). Together, these data suggest that the expression of *Esr1* is down-regulated by baicalin at the transcriptional level.

### Baicalin Activates Multiple Signaling Pathways Related to Tissue and Organ Morphogenesis in Basal Cells

**FIGURE 5:**
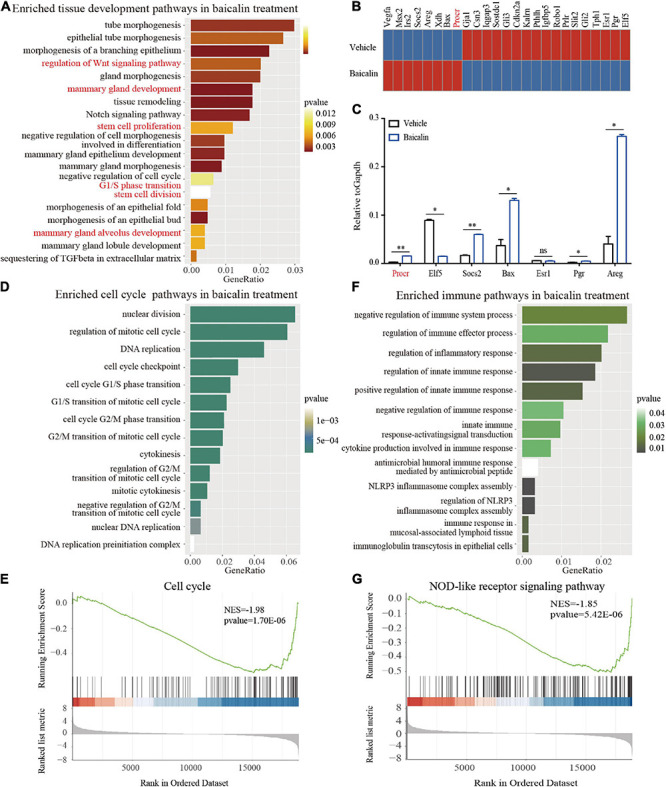
Transcriptome analysis of basal cells treated with baicalin. **(A)** GO enrichment analysis of development-related pathways in 3D-cultured basal cells. **(B)** Heatmap analysis of genes related to mammary gland development differentially regulated in response to baicalin. **(C)** RT-qPCR analysis indicates that baicalin regulates genes related to mammary gland development. Student’s *t*-test: **P < 0.01; *P < 0.05; ns, not significant. **(D)** GO enrichment analysis of cell division-related pathways in 3D-cultured basal cells. **(E)** GSEA (Gene Set Enrichment Analysis) revealed that cell cycle genes are down-regulated by baicalin. **(F)** GO enrichment analysis of immune response-related pathways in 3D-cultured basal cells. **(G)** GSEA revealed that NOD-like receptor signaling pathways are down-regulated by baicalin.

We found that baicalin can regulate the behavior of MaSCs and mammary development. To obtain a comprehensive understanding of the biological activity of baicalin, we analyzed the changes in the transcriptome of mammary basal cells induced by baicalin by performing RNA sequencing (RNA-seq). Gene ontology (GO) enrichment analysis revealed that baicalin activated multiple organ development-related genes, such as those involved in the Wnt signaling pathway, stem cell proliferation, stem cell division, and mammary gland alveolus development (Figure 5A). Heatmap analysis indicated that many critical genes, including *Procr*, *Areg*, *Elf5*, *Socs2*, and *Bax*, that are related to mammary gland development in basal cells were up-regulated after baicalin treatment (Figure 5B). The expression levels of these key genes were measured by RT-qPCR. The results indicated that the *Procr*, *Elf5*, *Socs2*, *Bax*, and *Areg* genes were significantly up-regulated in the baicalin-treated group, which validated the accuracy of the RNA-seq results. In addition, RT-qPCR results indicated that the expression levels of *Esr1* and *Pgr* in basal cells were extremely low, which further confirmed that the sorted cells used for sequencing were basal cells (Figure 5C). GO enrichment analysis revealed that baicalin activates cell division-related pathways (Figure 5D) and immune response-related pathways (Figure 5F). Gene set enrichment analysis (GSEA) of basal cells also confirmed the enrichment of cell cycle (Figure 5E) and immune response signaling pathways (Figure 5G) after baicalin treatment.

### Baicalin Exhibits Steroid Hormonal Bioactivities in Luminal Cells

**FIGURE 6:**
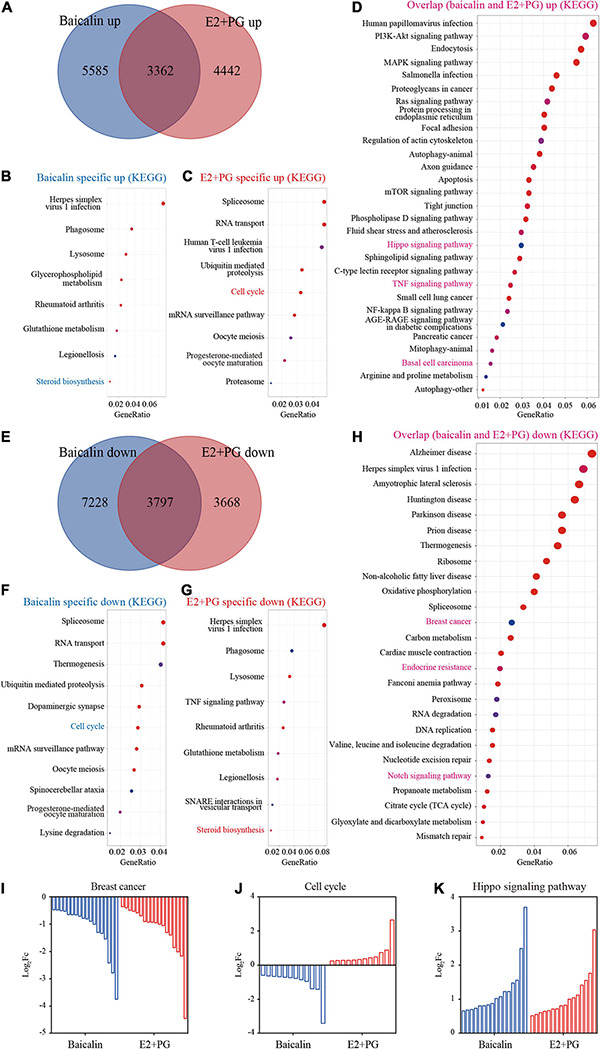
Transcriptome analysis of luminal cells treated with baicalin or E2 + PG. **(A)** Venn diagram showing the overlap of genes up-regulated in 3D-cultured luminal cells treated with baicalin or E2 + PG. **(B,C)** Enriched pathways from KEGG analysis of genes up-regulated in luminal cells treated with baicalin **(B)** and E2 + PG **(C)**. **(D)** Enriched pathways from KEGG analysis of genes in luminal cells up-regulated by both baicalin and E2 + PG. **(E)** Venn diagram showing the overlap of genes down-regulated in 3D-cultured luminal cells treated with baicalin or E2 + PG. **(F,G)** Enriched pathways from KEGG analysis of genes down-regulated in luminal cells treated with baicalin **(F)** and E2 + PG **(G)**. **(H)** Enriched pathways from KEGG analysis of genes in luminal cells down-regulated by both baicalin and E2 + PG. **(I)** Representative pathway (breast cancer) down-regulated by baicalin and E2 + PG. **(J)** Representative pathway (cell cycle) down-regulated in luminal cells by baicalin, but up-regulated in luminal cells by E2 + PG. **(K)** Representative pathway (Hippo pathway) up-regulated in luminal cells by baicalin and E2 + PG.

To further understand the underlying molecular mechanism, we compared the gene profiles induced by baicalin with those induced by hormones (E2 + PG). RNA-seq analysis indicated that nearly 40% of the genes up-regulated by baicalin and E2 + PG in 3D-cultured luminal cells overlapped (Figure 6A). We further analyzed the up-regulated genes using KEGG analysis and found that steroid synthesis-related genes were specifically up-regulated by baicalin (Figure 6B), cell cycle-related genes were specifically up-regulated by E2 + PG (Figure 6C), and Hippo signaling pathway-related genes were up-regulated by both baicalin and E2 + PG (Figure 6D). As to the down-regulated genes, more than 3,000 genes involved in breast cancer oncogenesis were down-regulated by both baicalin and E2 + PG (Figures 6E,H). Meanwhile, cell cycle-related genes were specifically down-regulated by baicalin (Figure 6F), and steroid biosynthesis-related genes were specifically down-regulated by E2 + PG (Figure 6G). Notably, breast cancer- and endocrine resistance-related genes were downregulated by baicalin (Figures 6H,I). It should be emphasized that cell cycle-related genes were up-regulated by E2 + PG and down-regulated by baicalin (Figure 6J). The up-regulation of Hippo signaling-related genes by both baicalin and E2 + PG indicates that baicalin and steroid hormones have similar effects on mammary development (Figure 6K). Taken together, these findings suggest that baicalin exhibits steroid hormone-like biological activities in the regulation of mammary development and breast cancer oncogenesis.

### Baicalin Suppresses Growth of Luminal Breast Cancer Tumors, but Promotes the Growth of TNBC Tumors

**FIGURE 7:**
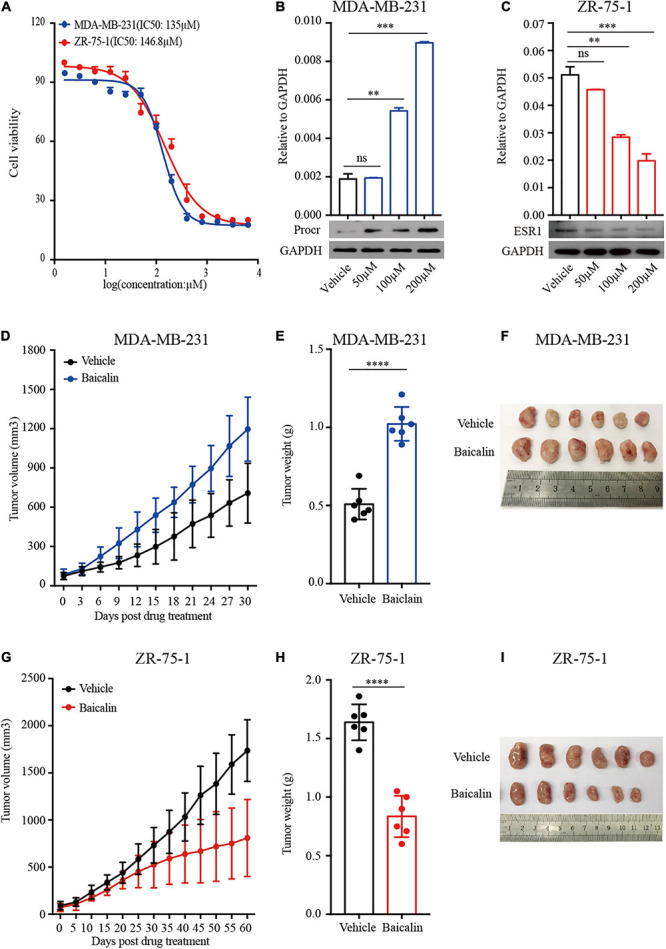
Baicalin promotes the growth of TNBC tumors and suppresses the growth of luminal breast cancer tumors. **(A)** An MTT assay was performed on MDA-MB-231 and ZR-75-1 cells treated with baicalin. **(B)** RT-qPCR and western blot analyses were performed to detect *PROCR* expression in MDA-MB-231 cells treated with baicalin. Data are presented as mean ± SD from three independent experiments. Student’s *t*-test: ^∗∗∗^*P* < 0.001; ^∗∗^*P* < 0.01; ns, not significant. **(C)** RT-qPCR and western blot analyses were performed to detect *ESR1* expression in ZR-75-1 cells treated with baicalin. Data are presented as mean ± SD from three independent experiments. Student’s *t*-test: ^∗∗∗^*P* < 0.001; ^∗∗^*P* < 0.01; ns, not significant. **(D)** Effect of baicalin on tumor volume in an MDA-MB-231 xenograft mouse model. The data are representative results obtained from more than five mice. **(E)** Effect of baicalin on tumor weight in an MDA-MB-231 xenograft mouse model. The data are representative results obtained from more than five mice. Student’s *t*-test: ^∗∗∗∗^*P* < 0.001. **(F)** A representative MDA-MB-231 tumor at the end of the experiment. **(G)** Effect of baicalin on tumor volume in a ZR-75-1 xenograft mouse model. The data are representative results obtained from more than five mice. **(H)** Effect of baicalin on tumor weight in a ZR-75-1 xenograft mouse model. The data are representative results obtained from more than five mice. Student’s *t*-test: ^∗∗∗∗^*P* < 0.001. **(I)** Representative ZR-75-1 tumor at the end of the experiment.

Given that baicalin regulates mammary cell proliferation and mammary development, we next studied the role of baicalin in the treatment of breast cancer. Two breast cancer cell lines, ZR-75-1 (luminal breast cancer subtype) and MDA-MB-231 (TNBC subtype), were used in our study. MTT analysis indicated that the IC50 values of baicalin in MDA-MB-231 and ZR-75-1 cells were 135 and 147 μM, respectively (Figure 7A). We then examined *PROCR* (a known breast cancer stem cell marker) (Wang et al., 2019) expression in MDA-MB-231 cells and *ESR1* expression in ZR-75-1 cells upon baicalin treatment. RT-qPCR and western blot analyses indicated that *PROCR* expression was up-regulated and *ESR1* expression was down-regulated in a dose-dependent manner after baicalin treatment (Figures 7B,C). Xenograft tumor model analysis indicated that baicalin significantly promoted tumor growth of the MDA-MB-231 cell line (Figures 7D–F), but significantly suppressed tumor growth of the ZR-75-1 cell line (Figures 7G–I), as determined by measuring tumor volume and tumor weight. Together, these data suggest that baicalin may have a therapeutic effect on the ZR-75-1 cell line, but not on the MDA-MB-231 cell line.

## Discussion

The development of mammary glands is driven by steroid hormones (Feng et al., 2007; Cai et al., 2014); thus, maintaining hormone homeostasis is vital for mammary gland development (Kolla et al., 2018). TCMs have been shown to mediate hormone secretion and regulate physiological homeostasis. For example, *S. baicalensis*, an important Chinese medicine, has been reported to have estrogen-like activity (Ruoguang Wang et al., 2003). However, the effective component of *S. baicalensis* and the molecular mechanism by which it regulates organogenesis are unclear. Baicalin is one of the key components of *S. baicalensis* (Wang G. et al., 2018). In this study, we investigated the effect of baicalin on the development of mammary glands *in vivo*, and the effect of baicalin on the proliferation of mammary gland primary cells *in vitro*. The results indicate that baicalin promotes mammary duct elongation at puberty and alveoli formation during pregnancy in a wild-type mouse model. In addition, the findings using a mouse model lacking endogenous hormones further demonstrate that baicalin induces duct growth and lateral branching. Since we performed unilateral resection in our ovariectomy model, the actual induction of baicalin on duct growth and lateral branching may be more significant. Here, we confirmed that baicalin can be used as a hormonal regulator to promote mammary development during puberty and pregnancy, providing a possible option for the treatment of patients with breast developmental defects.

To further explore baicalin’s function, *in vitro* 3D cultures of basal and luminal cells were used. The results demonstrated that baicalin promotes the self-renewal of MaSCs while inhibiting the ability of luminal cells to form colonies. Previously Zeng and Nusse (2010) established a 3D culture system for MaSC expansion using Wnt3a stimulation. In addition, Cai et al. (2014) established a luminal cell and basal cell co-culture system for MaSC expansion in the presence of E2 and PG. Compared with these previous culture systems, our system using a new mitogen for the expansion of MaSCs is less expensive and more conducive to clinical application.

Herbs, especially TCMs, are considered to have therapeutic or symptom-alleviating effects in a variety of diseases. An extensive survey indicated that a considerable number of cancer patients are treated with Chinese herbal medicines (Damery et al., 2011). Studies showed that herbal medicines exhibit anti-cancer activity in various tumors: the Chinese herbal medicine Qingyihuaji Formula is usually used as an anti-cancer agent in pancreatic cancer therapy (Mattei et al., 2013); Ginseng can be used as an anti-cancer agent in the treatment of colorectal cancer (Wang and Yuan, 2008); and Withaferin A can inhibit breast tumor growth (Nagalingam et al., 2014). So far, there are more than 60 herbal complexes being studied as anti-cancer reagents (Pei et al., 2015). The studies to date have mainly focused on processes such as the epithelial-mesenchymal transition (Chung et al., 2015), inflammation (Lin et al., 2014), fibrosis (Huang et al., 2016), and abortion (Chen et al., 2015). The results from our study using a breast cancer model showed that baicalin promoted tumor growth of the MDA-MB-231 cell line (TNBC subtype), while suppressing tumor growth of the ZR-751 cell line (luminal breast cancer subtype). These results from xenograft tumor models were consistent with the results from 3D-cultured mammary primary cells, and indicated that baicalin promotes the growth of basal cells and inhibits the growth of luminal cells. However, we didn’t detect an effect of baicalin on HER2-positive breast cancer. Therefore, the effect of baicalin on HER2-positive breast cancer is unknown. The function of baicalin should be investigated in more breast cancer cell lines. Taken together, our results show that TCMs should be precisely chosen according to the patient’s condition, otherwise they may be counterproductive.

To further understand the molecular mechanism, the signaling pathways in which baicalin participates in basal cells were investigated by transcriptome analysis. Interestingly, the enrichment of the mammary gland alveolus development pathway in basal cells was consistent with the increase in alveolar formation during pregnancy in the presence of baicalin. In addition, the enrichment of cell cycle and immune response signaling pathways after baicalin treatment is consistent with previous reports that baicalin triggers cell cycle arrest at the S phase (Gong et al., 2017) and suppresses inflammatory processes (Yang et al., 2019).

In the transcriptome analysis of luminal cells, breast cancer- and endocrine resistance-related genes were downregulated by baicalin, which was accordance with the inhibitory effect of baicalin on the ZR-751 cell line, suggesting that baicalin has the potential for application in the treatment of certain types of breast cancer. However, basal cell carcinoma genes were up-regulated by both baicalin and E2 + PG, which was accordance with the stimulatory effect of baicalin on the MDA-MB-231 cell line, indicating that baicalin should not be used for triple negative breast cancer patients. It should be emphasized that cell cycle-related genes were up-regulated by E2 + PG and down-regulated by baicalin, which may explain why baicalin inhibited the proliferation of luminal cells, while E2 + PG did not. The up-regulation of Hippo signaling-related genes by both baicalin and E2 + PG indicates that baicalin and steroid hormones have similar effects on mammary development. Taken together, these findings suggest that baicalin exhibits steroid hormone-like biological activities in the regulation of mammary development and breast cancer oncogenesis.

Our study demonstrated that baicalin can act as a growth factor promoting *in vitro* mammary stem cell expansion, and that baicalin administration can promote postnatal mammary development. Our study also indicated that baicalin has an inhibitory effect on ZR-751 cell line tumors. In addition, global signaling pathways activated by baicalin were further revealed through bioinformatics analysis. In all, our study not only validates the biological function of baicalin in an organ development model, but also reveals its underlying biological function through transcriptome analysis. Our research provides theoretical guidance for the application of the traditional Chinese medicine *S. baicalensis* in treatment of human diseases.

## Data Availability Statement

The datasets presented in this study can be found in online repositories. The names of the repository/repositories and accession number(s) can be found below: RNA-seq data can be accessed under GEO accession numbers GSE100664 and GSE166157.

## Ethics Statement

The animal study was reviewed and approved by the Animal Care and Use Committee of Wuhan University.

## Author Contributions

WC, LW, and CC designed the experiments. WC, WW, and LY performed the experiments. LY, FH, XZ, and LW analyzed the data. WC, QZ, and CC wrote the manuscript. All authors read and approved the final manuscript.

## Conflict of Interest

QZ was employed by the company Shenzhen Tyercan Bio-pharm Co., Ltd. CC was employed by the company Shenzhen Beike Biotechnology Co., Ltd. The remaining authors declare that the research was conducted in the absence of any commercial or financial relationships that could be construed as a potential conflict of interest.
